# Rumpel-Leede phenomenon in a hypertensive patient due to mechanical trauma: a case report

**DOI:** 10.1186/s13256-016-0950-3

**Published:** 2016-06-05

**Authors:** Adam Hartley, Phang B. Lim, Sajad A. Hayat

**Affiliations:** 1 Department of Cardiology, Imperial College Healthcare NHS Trust, London, W12 0HS UK; 2 Department of Cardiology, University Hospitals Coventry and Warwickshire NHS Trust, West Midlands, UK

**Keywords:** Diabetes mellitus, Mechanical trauma, Petechial rash, Rumpel-Leede phenomenon

## Abstract

**Background:**

In this report, we present an interesting case of a patient with Rumpel-Leede phenomenon, a rare occurrence that can result in significant delays in medical treatment. This phenomenon is characterized by the presence of a petechial rash that results from acute dermal capillary rupture. In our patient, it occurred secondary to raised pressure in the dermal vessels caused by repeated inflation of a sphygmomanometer cuff. Contributory factors in Rumpel-Leede phenomenon include prevalent conditions such as diabetes mellitus, hypertension, thrombocytopenia, chronic steroid use, antiplatelets, and anticoagulants.

**Case presentation:**

A 58-year-old Russian woman with diabetes and hypertension presented to our hospital with a non-ST elevation myocardial infarction, and she subsequently developed a petechial rash on her distal upper limbs. A vasculitic screen was performed, with normal results.

**Conclusions:**

Given the timing and distribution of the rash, it was felt that this was an example of Rumpel-Leede phenomenon in a susceptible individual. This is an important diagnosis to be aware of in patients with vascular risk factors presenting for acute medical care who subsequently develop a petechial rash.

## Background

Rumpel-Leede sign was first reported in 1909 by Theodor Rumpel and again in 1911 by Carl Stockbridge Leede. While treating patients with scarlet fever, they both noted petechiae on patients’ arms distal to where a tourniquet had been applied. Historically, the tourniquet test (or Rumpel-Leede capillary fragility test) was used to assess patients for thrombocytopenia and capillary fragility [1–4]. This sign is still clinically relevant in modern medicine.

## Case presentation

A 58-year-old woman presented to the accident and emergency department of our hospital with left-sided chest pain exacerbated by exertion. Associated symptoms included shortness of breath, nausea, vomiting, and diaphoresis. She had a background of type 2 diabetes mellitus, myocardial infarction, atrial fibrillation on warfarin, heart failure, and stage 4 chronic kidney disease. At presentation, an examination revealed raised blood pressure of 207/140 mmHg with clinical signs of decompensated congestive cardiac failure.

Her electrocardiogram demonstrated ST depression and T-wave inversion in the lateral leads, and her chest x-ray confirmed the clinical findings of pulmonary edema. Bedside echocardiography demonstrated moderate to severe left ventricular systolic dysfunction with left ventricular hypertrophy and a calcific but unobstructive aortic valve. Given her risk factors, particularly her past history of myocardial infarction, an acute coronary syndrome was suspected, which was later confirmed with a raised high-sensitivity troponin I. Other differential diagnoses considered included aortic dissection and malignant hypertension.

The patient was treated with aspirin, clopidogrel, fondaparinux, and a high-dose statin. To help settle her chest pain, intravenous glyceryl trinitrate (GTN) was also started, she was given diuretics to treat her heart failure, and coronary angiography was planned. Despite this, her blood pressure remained high; therefore amlodipine was added.

The patient improved clinically within a few hours of admission; however, the following morning the patient was noted to have a fine, petechial, non-blanching rash over the dorsum of her left hand and forearm, as well as over her right hand and forearm, although to a lesser extent. There was no evidence of rash on her face, trunk, or lower limbs.

**Fig. 1:**
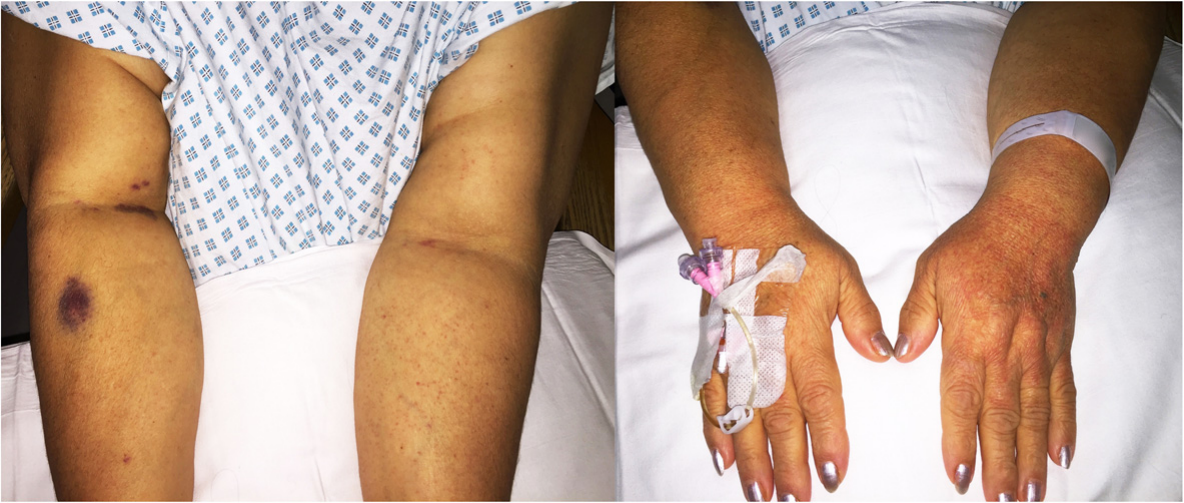
Bilateral petechial rash over the patient’s forearms and hands, more prominent on the left arm and hand

No features of meningism were present. Careful examination revealed the rash to be isolated to the areas shown in Fig. 1.

Owing to the petechial appearance of the rash, a vasculitic screen was requested. The appearance of the rash soon after admission raised the concern of a drug-related reaction; as such, clopidogrel and fondaparinux were withheld temporarily. The patient’s coronary angiography procedure was placed on hold pending the results of the vasculitic screen and to allow time to observe if the rash spread further or if the patient became systemically unwell.

The non-progressive nature of the rash over the following 24 hours and a clear, somewhat symmetrical demarcation below the level of the sphygmomanometer cuff raised the possibility of the Rumpel-Leede phenomenon due to acute dermal capillary rupture secondary to raised pressure in the dermal vessels with inflation of the sphygmomanometer cuff. The patient’s blood pressure had been regularly checked at 15-minute intervals after initiation of the GTN infusion. Initial measurements were done on the left arm, but later the sphygmomanometer cuff was switched to the right arm, at the patient’s request, due to pain because of the repeated high blood pressure readings in the first few hours.

Once our patient’s blood pressure was brought under control, her rash started to settle, and she went on to have successful stenting of her left anterior descending coronary artery with no adverse reaction to the reinstitution of clopidogrel. She experienced a 2-day delay in her treatment as a result of the rash. Greater awareness of this diabetic and hypertensive complication would have prevented this delay.

## Discussion

Today, the Rumpel-Leede sign may be observed iatrogenically in the context of continuous blood pressure monitoring, as was the case in our patient. The first reported association of Rumpel-Leede sign with hypertension and prolonged blood pressure monitoring was described by White *et al*. [5], and others have reported the same association [6]. Another example, recently described by Nguyen *et al*., includes “baby carrier purpura” caused by excessively tight baby carriers producing a tourniquet effect, resulting in capillary injury and a petechial rash [7].

Patients with diabetes mellitus or hypertensive vascular disease are susceptible to developing the Rumpel-Leede phenomenon. Long-standing diabetes mellitus can increase capillary fragility due to microangiopathy, and the hypertensive state leads to an increase of venous pressure, especially during blood pressure cuff inflations, as in our patient. In one study, 68 % of patients with diabetes demonstrated positive Rumpel-Leede phenomenon, as compared with 35 % in the control group. The longer duration of diabetes, as well as the presence of diabetic microvascular complications, favored the development of Rumpel-Leede sign [8].

Other contributory factors include thrombocytopenia, chronic steroid use, antiplatelets, and anticoagulants. Increased capillary fragility can also be seen in Ehlers-Danlos syndrome and other heritable connective tissue disorders [9]. As mentioned above, the tourniquet test has historically been used for the assessment of capillary fragility and thrombocytopenia [1–4], and even today it maintains its relevance, forming a useful component of the World Health Organization criteria for diagnosis of dengue fever [10].

## Conclusions

This case is an interesting example of a real-world problem seen in a patient with an acute medical presentation in whom early recognition of this rare phenomenon prevented costly delays and overinvestigation.
